# The New Secretary of Westminster Hospital

**Published:** 1921-05-07

**Authors:** Thomas Hayes

**Affiliations:** Clerk to the Governors. St. Bartholomew's Hospital, London, E.C. 1


### THE EDITOR'S LETTER-BOX.

*Correspondence on all subjects is invited, but we cannot in any way be responsible for the opinions expressed by our correspondents, who must give their name and address as a guarantee of good faith, but not necessarily for publication. Correspondents are reminded that brevity of style and conciseness of statement greatly facilitate early insertion.*

### The New Secretary of Westminster Hospital.

*To the Editor of* The Hospital.

Sir,—Will you allow me to correct an error in the notice of the appointment of a new Secretary of Westminster Hospital which appeared in your issue of the 16th inst.? You state that the gentleman appointed, Captain C. M. Power, has had about two years' experience of voluntary hospitals. Captain Power has been in the service of St. Bartholomew's Hospital for fifteen years. He entered as an assistant in the Appeal Department in 1905. In 1906 he was transferred to the Steward's Department, where he acted as registration clerk, and in 1912 was appointed an assistant in the clerk's office, where he remained until the outbreak of the war, when he was granted leave of absence and joined H.M. Forces. He was demobilised in May 1919, and two months later was appointed steward to the hospital. I am, Sir, your obedient servant,

Thomas Hayes,
Clerk to the Governors.

St. Bartholomew's Hospital, London, E.C. 1,
April 29, 1921.

